# 

**Published:** 1956-10

**Authors:** 


					WJ r7)
(1
y>
THE
MEDICAL JOURNAL
OF THE
SOUTH-WEST
Incorporating
The Bristol Mcdico-Chirurgical Journal
October 1956
VOL. 71 (iv). No. 262 Price 4/-

				

